# The metabolite ILA of *Akkermansia muciniphila* improves AP-related intestinal injury by targeting and inhibiting CASP3 activity

**DOI:** 10.3389/fmicb.2025.1669383

**Published:** 2025-12-05

**Authors:** Peiyu Li, Jiuliang Yu, Tao Li, Xiaoyong Gong, Le Li, Yi Cui, Jiayi He, Bo Li, Shuqi Wu, Qingyang Guan, Zhiming Zhang, Xingui Dai, Zhiwang Li

**Affiliations:** 1 The First Affiliated Hospital of Jinan University, Guangzhou, Guangdong, China; 2 Department of Gastroenterology, The First People’s Hospital of Chenzhou, Chenzhou, Hunan, China; 3 The First Affiliated Hospital of Xiangnan University, Chenzhou, Hunan, China; 4 Department of Critical Care Medicine, The First People's Hospital of Chenzhou, The ChenZhou Affiliated Hospital, Hengyang Medical School, University of South China, Chenzhou, Hunan, China; 5 Department of Critical Care Medicine, The First People's Hospital of Chenzhou, Chenzhou, Hunan, China; 6 Department of Pathology, The First People’s Hospital of Chenzhou, Chenzhou, Hunan, China; 7 Department of Pain Medicine, The First People’s Hospital of Chenzhou, Chenzhou, Hunan, China; 8 Department of Otorhinolaryngology Head and Neck Surgery, The First People’s Hospital of Chenzhou, Chenzhou, Hunan, China; 9 Department of Anesthesiology, The First People’s Hospital of Chenzhou, Chenzhou, Hunan, China

**Keywords:** ILA, gut microbiome, acute pancreatitis, intestinal injury, CASP3

## Abstract

**Objective:**

Acute Pancreatitis (AP) is a common acute abdominal disease in clinical practice. The gut microbiome is recognized as a key regulator in the development of acute pancreatitis. *Akkermansia muciniphila* (AKK) is recognized as a functional probiotic strain and has a beneficial effect on the progression of many diseases. However, the role of the AKK in the development of AP remains unclear. Here, we evaluated the potential contribution of AKK to AP.

**Design:**

Relative abundance of gut microbial AKK in AP was evaluated. A rat model of acute pancreatitis was established by retrograde pancreatic duct infusion of sodium taurocholate. Non-targeted and targeted metabolomics analysis were used for metabolites analysis.

**Results:**

We first found that the relative abundance of gut microbial AKK in AP patients was significantly reduced compared with that in healthy subjects. Live AKK supplementation, as well as supplementation with its culture supernatant, remarkably alleviates AP-related intestinal injury in AP rat models. Metabolomics studies found that the live AKK was able to generate Indole-3-lactic acid (ILA). ILA exerted a protective effect against AP-related intestinal injury, and significantly reduce inflammatory cell activation and pro-inflammatory factor overproduction. The mechanistic study revealed that ILA inhibits the apoptosis of intestinal epithelial cells by suppressing the activity of CASP3, and improves the role of intestinal barrier dysfunction in the AP model.

**Conclusion:**

We revealed that ILA, derived from live AKK, may act as a novel endogenous agonist for CASP3. ILA may serve as a new potential treatment method for intestinal injury in AP after successfully translating its efficacy into clinical practice.

## Introduction

Acute Pancreatitis (AP) is a common acute abdominal disease in clinical practice. In recent years, the global incidence of AP has shown a significant upward trend. Currently, the annual incidence rate has reached 13–45 cases per 100,000 people, causing a heavy medical burden worldwide (Goyal et al., 2017; Szatmary et al., 2022). AP-related intestinal injury, as one of the most clinically significant complications of AP, is a critical determinant of disease prognosis. The disruption of the integrity of the intestinal barrier can lead to translocation of bacteria and endotoxins, thereby triggering systemic inflammatory response syndrome and multiple organ dysfunction (Maheshwari and Subramanian, 2016; Zhang et al., 2023; Schietroma et al., 2016), which is also an important pathological basis for the persistently high mortality rate of AP patients. Intestinal flora dysbiosis delivers a “second hit” by promoting bacterial translocation and secondary infection during acute pancreatitis. Studies have shown that intestinal flora imbalance affects the etiology and severity of AP through the disruption of the intestinal barrier, local or systemic inflammatory responses, bacterial translocation, and the regulatory effects of microbial metabolites. The traditional treatment regimens for AP-related intestinal injury still have significant limitations, especially in the insufficient clinical response rate of anti-inflammatory intervention and mucosal repair strategies. The latest evidence indicates that this therapeutic dilemma may be closely related to the high heterogeneity of the composition of the gut microbiota among individuals and its complex interaction with the host immune system (Zhang et al., 2023). Recent evidence suggests that alterations in gut microbial composition and metabolites such as phytosphingosine can predispose individuals to metabolic dysfunction, potentially aggravating lipid-mediated pancreatitis (Li et al., 2025). Screening probiotic strains that are both safe and enhance intestinal barrier function is a topic worthy of discussion in the field of treatment and prevention of AP-related intestinal injury.

*Akkermansia muciniphila* (AKK), as a strict gram-negative anaerobic bacterium, has become a star strain in the field of immune-metabolic axis regulation due to its unique ability to regulate the dynamic balance of the mucus layer of the intestines (Zhai et al., 2019; Zhao et al., 2024). In the field of metabolic diseases, AKK significantly improves insulin resistance by activating the intestinal FIAF/AMPK signaling pathway. Its role in regulating the Browning of white adipose tissue has been verified by mechanism in multiple human cohort studies and animal experiments (Entezari et al., 2022; Jung et al., 2018). It is worth noting that Professor Chen Peng’s team revealed a novel tripeptide Arg- Lys- His (RKH), from live AKK. The compound exhibits beneficial phenotypes in bacterial infectious sepsis models by specifically inhibiting inflammatory responses (Xie et al., 2023). However, whether AKK regulates tight junction remodeling through the communication mechanism with intestinal epithelium and mediates the protective effect of intestinal barrier function in the process of intestinal barrier injury induced by AP, remains a scientific proposition to be clarified.

This study reveals a new mechanism by which AKK protects intestinal barrier function in the AP rat model. Through the clinical cohort of AP patients, we observed that the intestinal microbiota of AP patients showed characteristic depletion of AKK abundance, and this change was significantly negatively correlated with the severity of the disease. Mechanism studies have found that the specific metabolite of AKK, Indole-3-lactic acid (ILA), inhibits the apoptosis of intestinal epithelial cells by suppressing the activity of CASP3, and improves the role of intestinal barrier dysfunction in the AP model. This discovery not only expands the pathophysiological function of AKK, but also provides a new potential treatment approach for the clinical treatment of AP-related intestinal barrier dysfunction.

## Materials and methods

### Research ethics approval

All animal experiments strictly followed the “Guidelines for the Care and Use of Laboratory Animals” of the National Institutes of Health (NIH) of the United States and were approved by the Guangzhou Miles Biosciences Application for Laboratory Animal (Approval Numbers: IACUC- MIS2023053). Studies involving human bodies have all been reviewed and approved by the Medical Ethics Committee of the First People’s Hospital of Chenzhou (Approval Numbers: 2025005).

### Human sample

This study included a total of 24 AP patients and 19 healthy controls (detailed information in Supplementary Table 1). The collected plasma and fecal samples were stored at −80 °C for future use. Criteria for inclusion included meeting the Modified Atlanta Criteria for diagnosis of AP within 24 h after onset of the disease, the age ranges from 18 to 80. The exclusion criteria included transferred from other institutions, acute attack of chronic pancreatitis, combined with severe organ dysfunction, such as renal failure, chronic heart disease, etc., suffering from autoimmune diseases, malignant tumors or blood system diseases, pregnancy or lactation, pancreatitis after trauma, surgery, tumor or endoscopic retrograde cholangiopancreatography (ERCP).

Patients’ samples were collected and analyzed. But patients and the public were not involved in the design, conduct, reporting or dissemination plans of this research.

### Rat model

The study used 6-8-week-old SD rats weighing 200-220 g, which were purchased from the Animal Center of Southern Medical University. All animal experiments were approved by the Animal Ethics Center of Guangzhou Miles Biosciences Application for Laboratory Animal and complied with the national and international guidelines for the Care and Use of Laboratory Animals.

AP rats was induced via a retrograde infusion of 5% sodium taurocholate into the pancreatic duct (a classic model of acute pancreatitis) (Schmidt et al., 1992). The animals were anaesthetized with sodium pentobarbital (50 mg/kg, Sigma-Aldrich), and the abdomen was opened via a midline incision. First, we identified the common bile duct and duodenum. The pancreatic bile duct was occluded with two microvascular clamps to prevent reflux. In total, 5% sodium taurocholate was injected into the pancreatic duct at a dose of 0.1 mL/100 g body weight (at an injection velocity of 0.1 mL/min). Then, the injection site was pressed for 3 min. Successful model induction was confirmed by visual observation of the pancreas turning dark red. Subsequently, the puncture needle was removed, the vascular clamp was loosened, and sutured the duodenal wall of the puncture. The abdomen was closed layer by layer to complete the operation. In the Sham-operated group, the incisions were closed immediately after turning over the pancreas. Mice were euthanized by cervical dislocation 24 h after the operation for detection of systemic inflammation and organ damage.

Safety assessment of ILA in rats was performed. Rats were randomly assigned into two groups, including the ILA group and the vehicle group. Two groups of rats were gavaged with ILA (50 mg/kg) or saline for three consecutive days. Thereafter, the rats were euthanized for the purpose of evaluating hepatic and renal function (Xie et al., 2023).

ILA administration protocols (dosage, route, frequency): Animal experiment: ILA was administered (50 mg/kg, dissolved in 5% DMSO) by gavage daily for 1 week before establishing the model. All the rats had free access to water and food. Then rats were housed in a temperature- controlled colony room on a 12/12-h light–dark cycle (Percie du Sert et al., 2020).

### Fecal microbiota transplantation (FMT) experimental procedures

Donor and recipient: The feces of AP patients were collected. The abundance of AKK was detected by qPCR. The feces were divided into the high AKK group and the low AKK group, and saved them for future use. Recipient rats (*n* = 5-6/group) were pseudo-sterile rats pretreated with an antibiotic mixture (amoxicillin + vancomycin + neomycin) for 5 days.

Fecal Microbiota Transplantation (FMT) experimental procedures: The feces (200 mg) of AP patients were homogenized under anaerobic conditions using PBS (containing 10% glycerol), and then centrifuged (500 × g for 5 min) to obtain the supernatant. The receptor rats were administered the donor bacterial solution (or the control PBS) by gavage (200 μL per time, for 5 consecutive days).

AKK replenishment methods: Animal experiment: The standard strain of AKK (ATCC BAA-835), 10⁹ CFU (100 μL PBS suspension) was administered by gavage daily for 5 days before modeling was initiated.

### 16S rDNA gene sequencing

After collecting fecal samples from AP model rats, Microbial DNA was extracted from the feces, and then the hypervariable region V3V4 of the bacterial 16S rDNA gene was amplified with primer by an ABI GeneAmp 9,700 PCR thermocycler. An Illumina MiSeq (PE300) was used for sequencing, and the data were analyzed by Majorbio Bio. Then the optimized sequences were clustered into operational taxonomic units (OTUs) using UPARSE 11 with a 97% sequence similarity level. The most abundant sequence for each OTU was selected as a representative sequence. The taxonomy of each OTU representative sequence was analyzed by RDP Classifier against the 16S rDNA gene database (e.g., Silva v138) using a confidence threshold of 0.7. Mothur V.1.30.2 was used to calculate the alpha diversity including the Chao index, Shannon index and Simpson index based on the OTUs information. Then principal coordinate analysis (PCoA) based on bray- curtis dissimilarity was calculated with the Vegan V.2.4.3 package.

### Detection of serum biochemical indicators

Venous puncture was performed to collect blood into a blood collection tube containing EDTA. Plasma was obtained by centrifuging at 12,000 rpm for 10 min at 4 °C. The levels of IFN-*β*, IL-6, TNF-*α*, D-lactic acid, DAO and Endotoxin were determined using commercial kits as per the instructions.

### LC–MS analysis

The AKK supernatant was prepared and freeze-dried. Taken 20 milligrams of the sample and mix it with a specific proportion of methanol-aqueous solution (containing the internal standard 2-chloro-L-tryptophan), and homogenized at −10 °C. Next, ultrasonic extraction was carried out on ice, and then the supernatant was obtained through centrifugal separation. Finally, taken an appropriate amount of the supernatant for liquid chromatography-mass spectrometry/mass spectrometry (LC–MS/MS) analysis.

### Histopathological examination

The small intestinal tissue was fixed in 4% paraformaldehyde solution, embedded in paraffin, and cut into 5 μm. The sections were stained with H&E to distinguish the morphological structures of the normal and pathological tissues. The assessment of small intestinal injury included thickening of the intestinal vesicle wall, fusion, hemorrhage, white blood cell infiltration and tissue exudation, etc.

### Bacterial load analysis

The peritoneal lavage fluid of rats was collected 12 h after AP. The sample diluent was prepare, inoculated onto blood AGAR plate, and calculated the colony-forming units (CFU) after incubation. The results were expressed as CFU/ml (liquid samples).

### Drug affinity responsive target stability assay (DARTS)

Human Intestinal Epithelial Cells (HIEC) lysates were prepared and diluted to 5 mg/mL, then incubated with or without 20 mM ILA at room temperature for 1 h. Pronase was added to the samples at a dilution of 1:333 (w/w) for 30 min, then protein stability was analyzed using western blotting.

### Cellular thermal shift assay

To conduct the cellular thermal shift assay (CETSA) experiments, the harvested cell suspensions underwent three cycles of freeze–thaw using liquid nitrogen followed by centrifugation at 20000 × g for 20 min at 4 °C. The supernatant collected after centrifugation was divided into two aliquots: one treated with ILA and the other treated with an equal volume of DMSO. Incubated these aliquots for 2 h at room temperature. After treatment, each lysate was further divided into 10 aliquots and subjected to heating at different temperatures for 3 min each, followed by a 3-min cooling period at room temperature. To separate the cell debris, the lysates were centrifuged at 15000 × rpm for 15 min at 4 °C. The resulting supernatants were boiled by adding 5 × loading buffer and then analyzed through western blotting.

### Molecular docking analysis

The crystal structure of CASP3 was obtained from the Protein Data Bank (PDB ID: 5YQM). The chemical structure of ILA was downloaded from the PubChem database^1^. The structure-based modeling was performed in Schro¨ dinger-Maestro software (version 11.1).

### Statistical analysis

Unless otherwise specified, data in this study were expressed as mean ± SEM. The experimental data were calculated by the two-tailed unpaired Student’s *t*-test or one-way analysis of variance (ANOVA). The two-way ANOVA was used for statistical analysis in cellular thermal shift assay (CETSA). ANCOM method was used for statistical analysis in the relative abundance of AKK from rats feces. Wilcoxon rank-sum test was used for fecal microbiota analysis at the genus levels. Specific statistical methods were noted in the figure legends. The statistics of all data in this study were analyzed by GraphPad Prism V.6.02. A *p*-value of less than 0.05 (*P < 0.05, **P < 0.01, ***P < 0.001, ****P < 0.0001) was considered statistically significant. Other materials and methods are shown in Supplementary Files.

## Results

### AKK in the intestine is involved in the progression of AP in rats and patients

**Figure 1:**
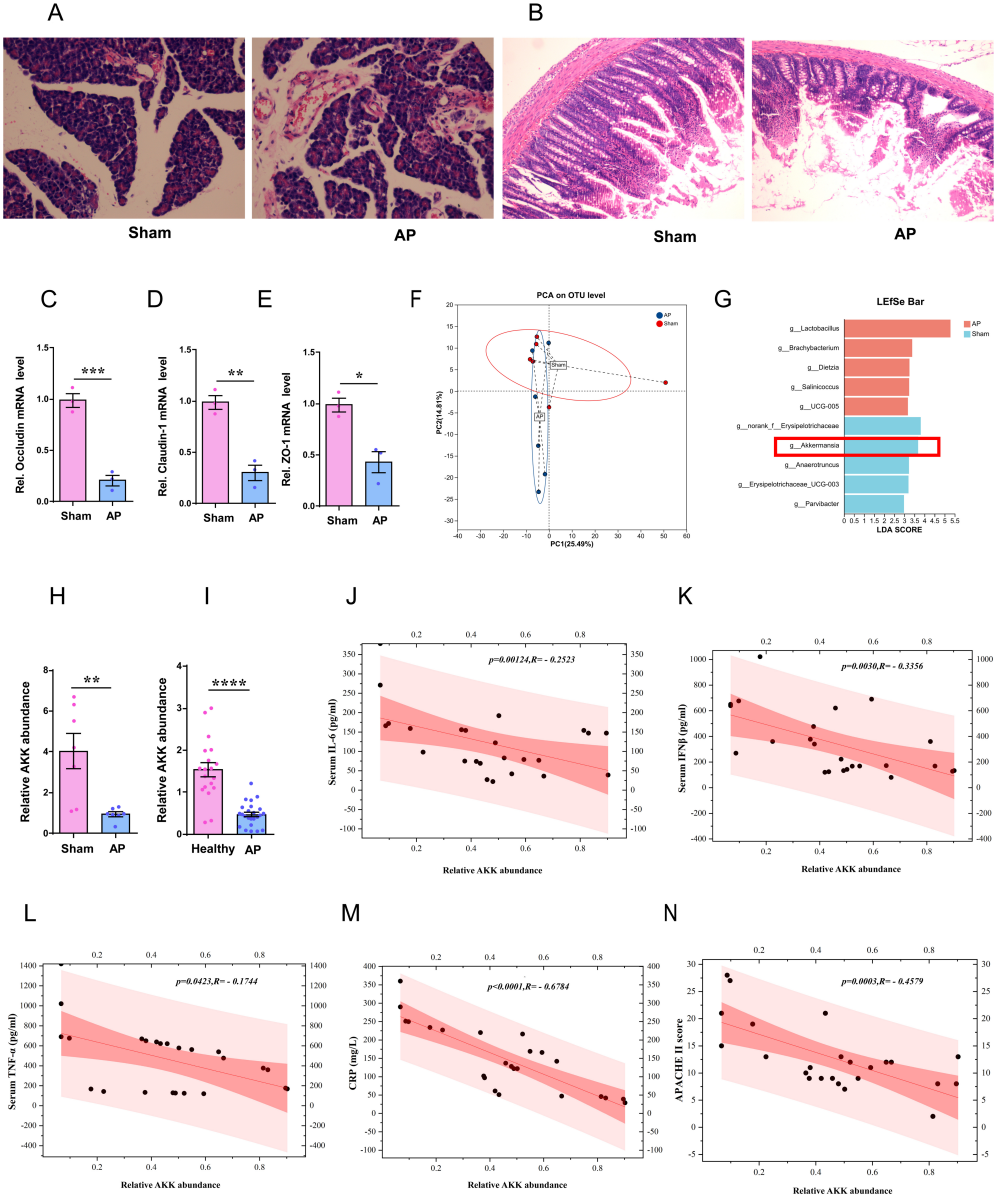
The abundance of AKK in the feces of AP model rats and AP patients decreased. **(A,B)** H&E staining images of rat pancreatic and small intestinal tissues, comparing the sham and acute pancreatitis (AP) groups. **(C-E)** Bar graphs show mRNA levels of tight junction proteins Occludin **(C)**, Claudin-1 **(D)**, and ZO-1 **(E)** in rat small intestinal tissues (*n* = 5). **(F)** PCA plot displays OTU clustering between groups. **(G)** LEfSe bar graph highlights differentially abundant taxa. **(H)** Bar graph shows fecal relative abundance of AKK in rats (*n* = 7). **(I)** Bar graph compares fecal AKK relative abundance between healthy controls (*n* = 19) and AP patients (*n* = 24). **(J-N)** Scatter plots illustrate Spearman correlation between fecal AKK abundance and plasma IL-6 **(J)**, IFN-β **(K)**, TNF-α **(L)**, CRP **(M)** levels, and APACHE II scores **(N)** in AP patients (*n* = 24).

In the study, the rat AP model was established by retrograding infusion of sodium taurocholate. Pathological examination revealed extensive glandular edema and interstitial exudation in the pancreatic tissue of the AP group (Figure 1A). The structure of the small intestinal mucosa was severely damaged, manifested as mucosa-lamina propria separation and villous erosion and destruction (Figure 1B). Quantitative PCR (qPCR) detection showed that the mRNA expression levels of tight junction proteins Occludin, Claudin-1 and ZO-1 in small intestinal tissues were significantly lower than those in the control group (*p < 0.05*, Figures 1C–E), suggesting the successful construction of the rat AP model. Secondly, we conducted 16S rDNA sequencing on fecal samples from rats in the Sham operation group (Sham) and the AP group (AP) to explore the potential association between the intestinal microbiota and the occurrence and development of AP. The results of principal coordinate analysis (PCA) showed that there was a significant separation of fecal microbiota composition at the genus level between the two groups, suggesting that the intestinal microbiota structure of AP rats had changed (Figure 1F). Linear discriminant analysis (LDA) showed that the abundance of the AKK genus in the feces of rats in the AP group was significantly reduced, while it was relatively abundant in the Sham group, highlighting the beneficial effects of the AKK genus (Figure 1G). Quantitative PCR (qPCR) further verified the decrease in the relative abundance of AKK in the feces of rats in the AP group (Figure 1H). These findings provided important insights for further exploration of AKK in the pathogenesis of AP in rats.

To deeply explore the potential role of AKK in patients with AP, we collected feces from 24 AP patients and 19 healthy subjects. Through quantitative PCR (qPCR) technology, we found that the relative abundance of AKK in the feces of AP patients was significantly lower than that of healthy subjects (Figure 1I), and the relative abundance of AKK in the feces of AP patients was negatively correlated with the levels of plasma inflammatory factors (Figures 1J–L), C-reactive protein (Figure 1M), and the severity of APACHE II score (Figure 1N) in the patients. The above results suggested that the exhaustion of AKK in the intestines of patients with AP may be an important cause of AP-related intestinal injury. (The clinical information of 24 patients with AP and 19 healthy subjects is presented in Supplementary Table S1).

### AKK alleviate AP-related intestinal injury by maintaining intestinal barrier function

**Figure 2:**
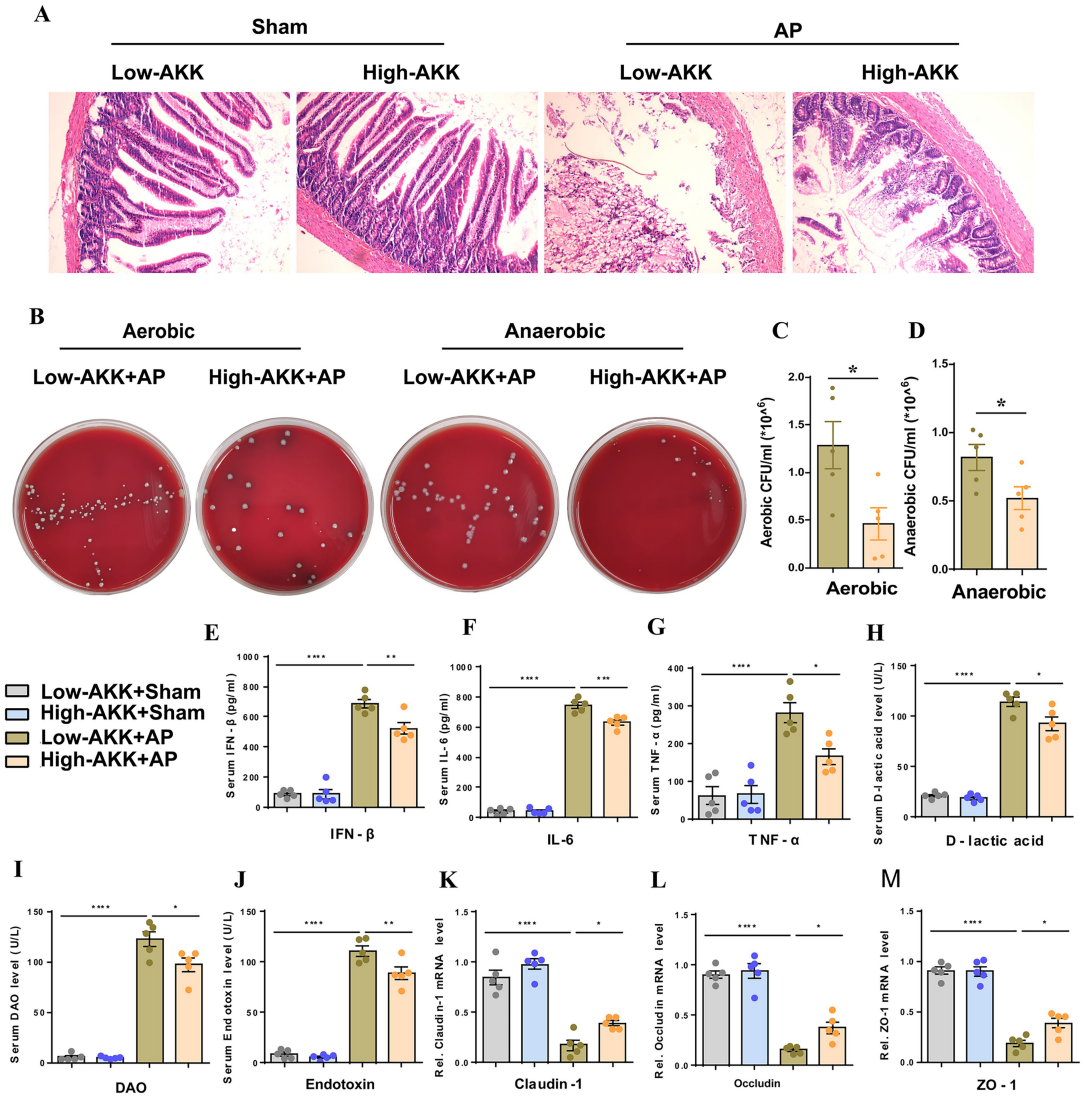
AKK improve intestinal barrier function and alleviate AP-related intestinal injury. **(A)** H&E staining of intestinal sections from sham and AP-treated rats, with low and high doses of AKK therapy (*n* = 5). **(B)** Representative images of bacterial cultures on petri dishes under aerobic and anaerobic conditions. **(C,D)** Bar graphs show quantitative analysis of bacterial loads (colony-forming units, CFU) in peritoneal lavage fluid (PLF) under aerobic **(C)** and anaerobic **(D)** conditions (*n* = 5). **(E-J)** Bar graphs display serum levels of IFN-β **(E)**, IL-6 **(F)**, TNF-α **(G)**, D-lactic acid **(H)**, DAO **(I)**, and endotoxin **(J)** (*n* = 5). **(K-M)** The mRNA levels of Claudin-1 **(K)**, Occludin **(L)**, and ZO-1 **(M)** of the small intestinal tissues.

To clarify the role of AKK in the progression of AP, we screened out the quarter-sample with the highest and lowest AKK abundance from 24 AP patients and divided them into the high AKK group and the low AKK group (*n* = 6 in each group). Subsequently, we transplanted these two groups of human feces into the recipient rats and constructed the AP model. Compared with the sham operation group, the small intestinal structure of rats in the low AKK + AP group was significantly damaged, while high AKK could significantly alleviate these pathological changes (Figure 2A). Compared with the low AKK + AP, the levels of plasma IFN-*β*, IL-6, TNF-*α*, D-lactic acid, DAO and Endotoxin in the high AKK + AP group of rats were all lower than those in the low AKK + AP group (Figures 2E–J). Meanwhile, we also collected the peritoneal lavage fluid of rats for culture and found that the number of bacterial colonies in the peritoneal lavage fluid of rats in the high AKK + AP group was significantly lower than that in the low AKK + AP group (Figures 2B–D). Further qPCR indicated that the mRNA expression levels of tight junction proteins (ZO-1, Occludin, Claudin-1) in the intestinal tissues of rats in the high AKK + AP group were significantly higher than those in the low AKK + AP group (Figures 2K–M). In conclusion, intestinal AKK may alleviate intestinal damage and microbiota translocation caused by AP by maintaining intestinal barrier function.

### Live AKK alleviates AP-related intestinal injury

**Figure 3:**
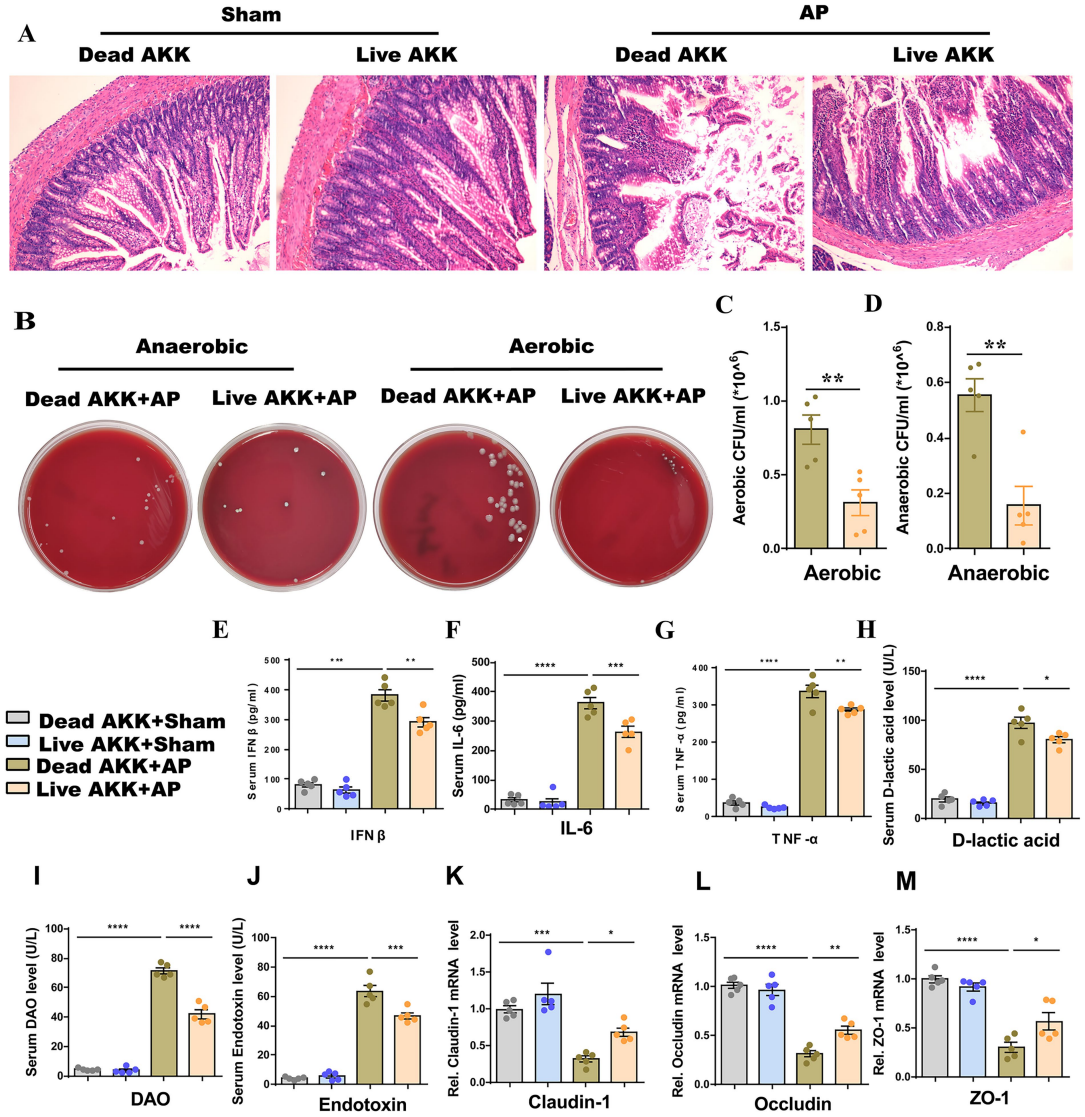
Live AKK improve AP-related intestinal injury. **(A)** H&E staining of intestinal sections from sham and AP-treated rats, with Dead and Live AKK (*n* = 5). **(B)** Representative images of bacterial cultures on petri dishes under aerobic and anaerobic conditions. **(C,D)** Bar graphs show quantitative analysis of bacterial loads (colony-forming units, CFU) in peritoneal lavage fluid (PLF) under aerobic **(C)** and anaerobic **(D)** conditions (*n* = 5). **(E-J)** Bar graphs display serum levels of IFN-β **(E)**, IL-6 **(F)**, TNF-α **(G)**, D-lactic acid **(H)**, DAO **(I)**, and endotoxin **(J)** (*n* = 5). **(K-M)** The mRNA levels of Claudin-1 **(K)**, Occludin **(L)**, and ZO-1 **(M)** of the small intestinal tissues.

To clarify the direct effect of AKK on AP, we pretreated rats with live AKK or heat-dead AKK for 5 days and then performed Sham operation or AP operation. Compared with dead AKK + AP, rats in the live AKK + AP group presented the following characteristics: The degree of damage to the small intestinal structure was reduced (Figure 3A), and the bacterial load in the peritoneal lavage fluid was significantly decreased (Figures 3B–D). The mRNA expressions of tight junction proteins (ZO-1, Occludin, Claudin-1) in small intestinal tissues were significantly upregulated (Figures 3K–M). The plasma levels of IFN-*β*, IL-6, TNF-*α*, D-lactic acid, DAO and Endotoxin in the Live AKK + AP group were significantly lower than those in the dead AKK + AP group (Figures 3E–J).

### Live AKK produce active metabolites that alleviate AP-related intestinal injury

**Figure 4:**
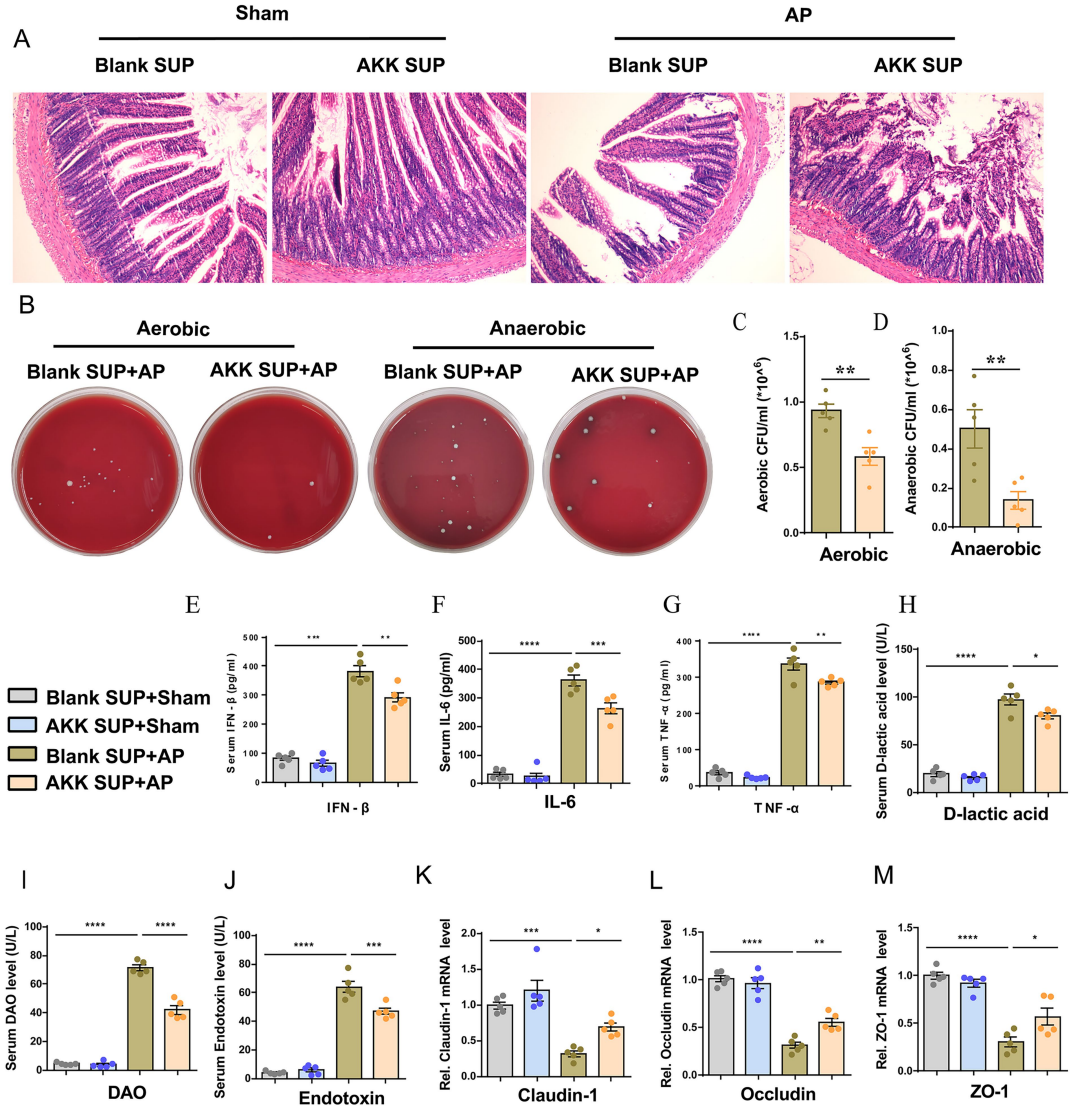
Live bacteria AKK produce active metabolites that alleviate AP-related intestinal injury. **(A)** H&E staining of intestinal sections from sham and AP-treated rats, with blank and live AKK supernatants (*n* = 5). **(B)** Representative images of bacterial cultures on petri dishes under aerobic and anaerobic conditions. **(C,D)** Bar graphs show quantitative analysis of bacterial loads (colony-forming units, CFU) in peritoneal lavage fluid (PLF) under aerobic **(C)** and anaerobic **(D)** conditions (*n* = 5) **(E–J)** Bar graphs display serum levels of IFN-β **(E)**, IL-6 **(F)**, TNF-α **(G)**, D-lactic acid **(H)**, DAO **(I)**, and endotoxin **(J)** (*n* = 5). **(K-M)** The mRNA levels of Claudin-1 **(K)**, Occludin **(L)**, and ZO-1 **(M)** of the small intestinal tissues.

The above data suggest that only live AKK can alleviate intestinal damage and microbiota translocation caused by AP. We speculate that the protective effect of AKK may depend on the bioactive substances produced by the live bacteria. To verify our hypothesis, we evaluated the intervention effect of the supernatant of live AKK culture on AP. Compared with the blank supernatant treatment group, the degree of small intestinal structure destruction in AP rats pretreated with the supernatant of live AKK culture was reduced (Figure 4A), and the bacterial load in peritoneal lavage fluid was significantly decreased (Figures 4B–D). The levels of plasma IFN-β, IL-6, TNF-α, D-lactic acid, DAO and Endotoxin were all decreased (Figures 4E–J). The mRNA expressions of tight junction proteins (ZO-1, Occludin, Claudin-1) in the small intestinal tissues of the live AKK culture rat group were significantly upregulated (Figures 4K–M). This result indicates that the supernatant of live bacterial AKK contains active metabolites that can alleviate intestinal damage and bacterial translocation caused by AP.

### The metabolite ILA of AKK can alleviate AP-related intestinal injury

**Figure 5:**
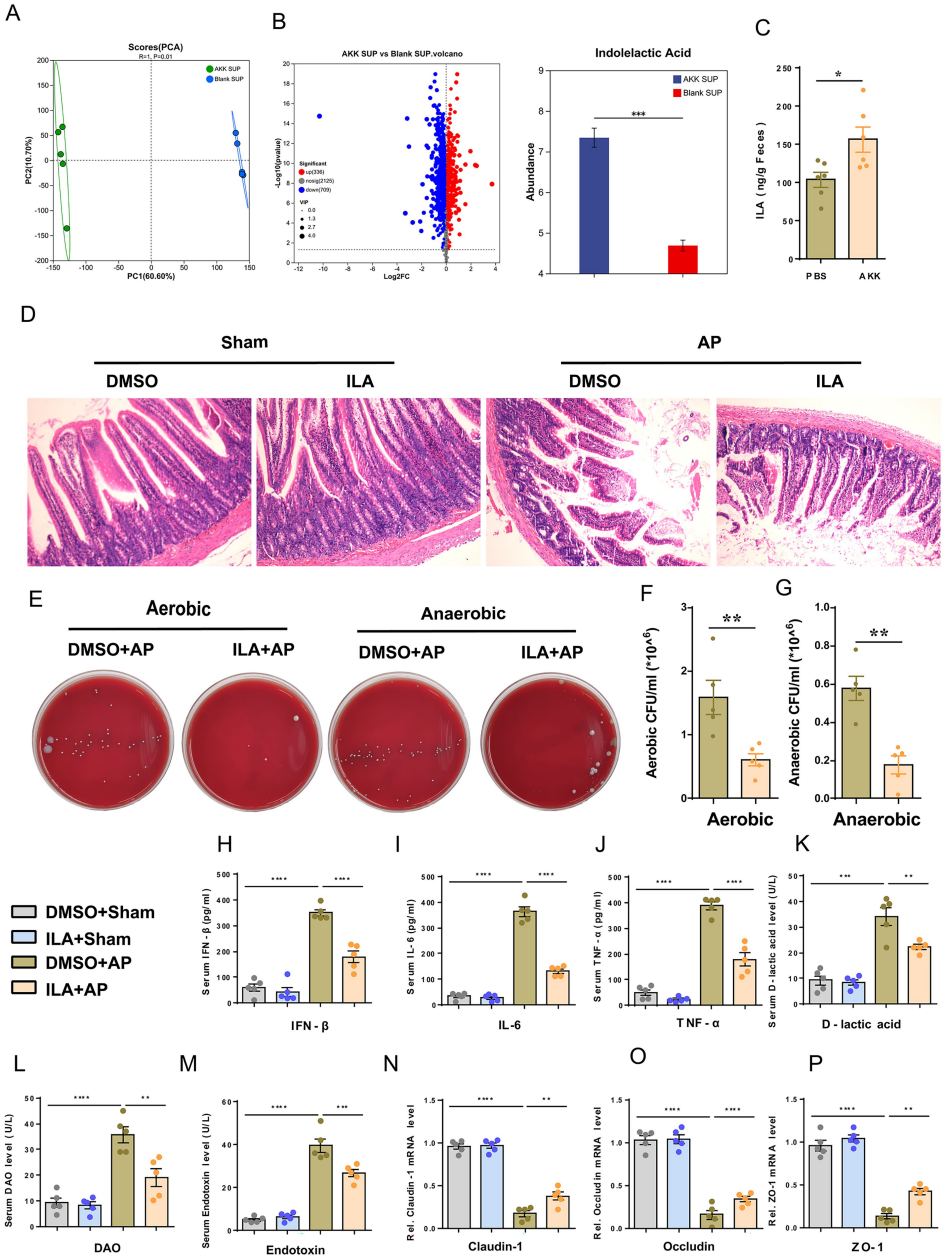
ILA alleviates AP-related intestinal injury. **(A)** Scatter plots of PCA of non-targeted metabolomics analysis from blank and live AKK supernatants (*n* = 5). **(B)** Volcano plot of non-targeted metabolomics analysis (AKK group vs blank group). **(C)** ILA concentration in cecum contents of germ-free (GF) rats gavaged with live AKK or vehicle (*n* = 5). **(D)** H&E staining of intestinal sections from sham and AP-treated rats, with blank and live AKK supernatants (*n* = 5). **(E)** Representative images of bacterial cultures on petri dishes under aerobic and anaerobic conditions. **(F, G)** Bar graphs show quantitative analysis of bacterial loads (colony-forming units, CFU) in peritoneal lavage fluid (PLF) under aerobic. **(F)** and anaerobic **(G)** conditions (*n* = 5). **(H–M)** Bar graphs display serum levels of IFN-β. **(H)**, IL-6 **(I)**, TNF-α **(J)**,D-lactic acid **(K)**, DAO **(L)**, and endotoxin **(M)** (*n* = 5). **(N-P)** The mRNA levels of Claudin-1 **(N)**, Occludin **(O)**, and ZO-1 **(P)** of the small intestinal tissues.

To reveal the protective active substances secreted by live AKK, in this study, non-targeted metabolomics analysis was conducted on the blank medium and the supernatant of live AKK culture. Principal component analysis (PCA) and volcano plot showed that there were significant differences between the two groups of metabolic profiles. Notably, ILA is specifically enriched in the supernatant of live AKK (Figures 5A,B). Further verification by targeted metabolomics showed that the ILA level in the supernatant of live AKK was significantly higher than that in the blank medium (*p* < 0.001, Figure 5C).

We further explored the potential therapeutic value of ILA in AP, and prior to AP surgery, rats were administered ILA (50 mg/kg, dissolved in 5% DMSO) via gavage for three consecutive days. ILA did not cause any impairment to liver and kidney functions in the rats, followed by sham or AP surgery (Supplementary Figure S1). The results showed that, compared with the DMSO+AP group, the degree of small intestinal structure destruction in rats of the ILA + AP group was reduced (Figures 5D), and the bacterial load in the peritoneal lavage fluid was significantly reduced (Figures 5E–G). The levels of plasma IFN-*β*, IL-6, TNF-*α*, D-lactic acid, DAO and Endotoxin in the ILA + AP group rats were all decreased (Figures 5H–M), and the mRNA expressions of tight junction proteins (ZO-1, Occludin, Claudin-1) of the small intestinal tissues were significantly upregulated (Figures 5N–P). These findings suggest that ILA may be a potential metabolite of AKK in alleviating AP-related intestinal injury.

### ILA targets CASP3 to inhibit apoptosis of human intestinal epithelial cells (HIEC)and alleviate AP-related intestinal injury

**Figure 6:**
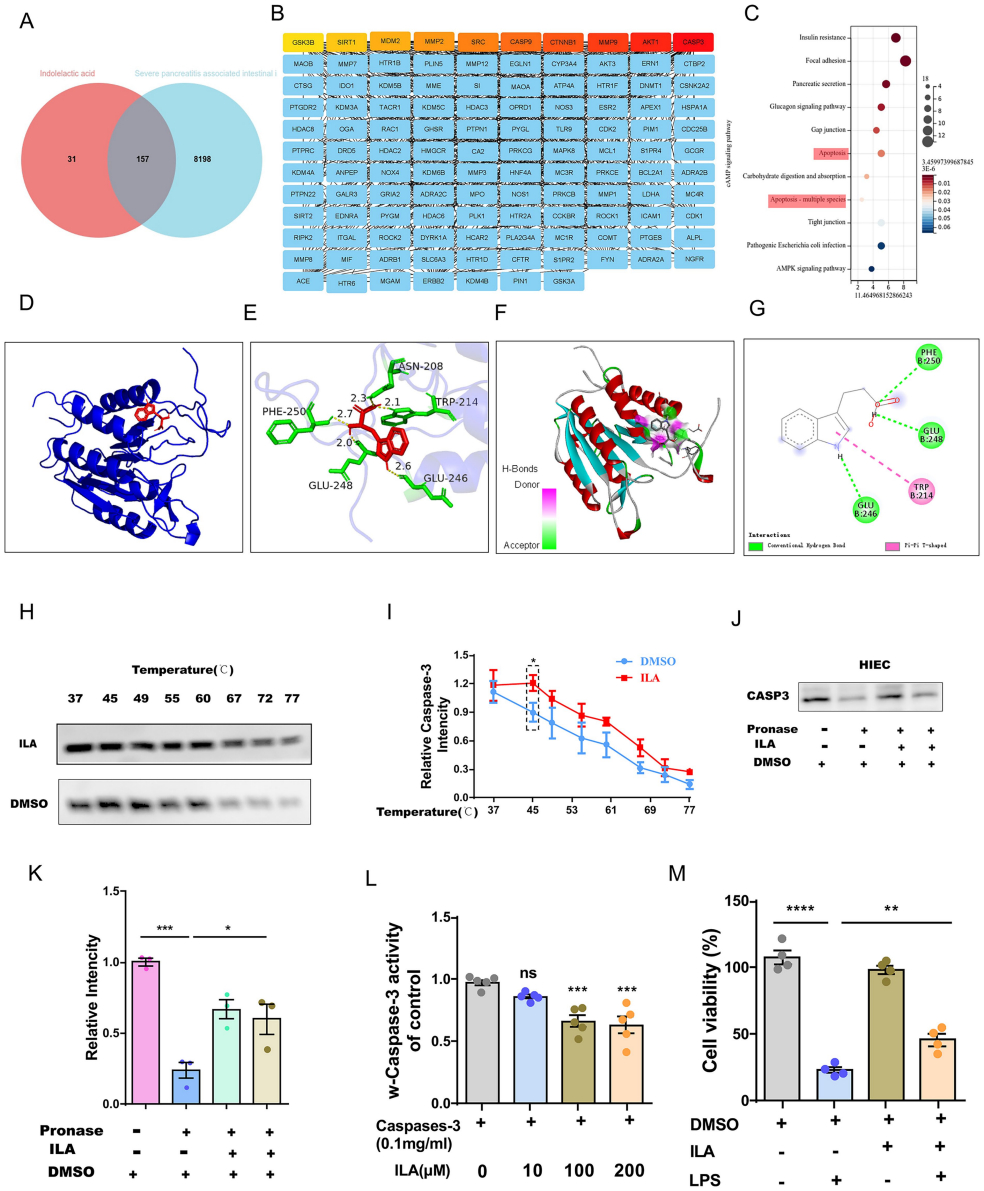
ILA alleviates intestinal injury by directly targeting CASP3 and inhibiting apoptosis. **(A)** Venn diagram showing overlapping genes between ILA (indolelactic acid) potential targets and AP-related intestinal injury genes. (B) List of the overlapping genes from the Venn diagram. (C) KEGG pathway analysis bar chart showing the common targets are significantly enriched in the Apoptosis and Focal Adhesion pathways. **(D-G)** Molecular docking models predict the binding conformation between ILA and the CASP3 protein. **(H,I)** DARTS assay validates the binding of ILA to CASP3, showing protein stability. **(J,K)** CETSA further confirms the ILA-CASP3 interaction by demonstrating thermal stability shift. **(L)** Bar graph shows ILA concentration-dependently inhibits Caspase-3 activity in human intestinal epithelial cells (HIECs), using Raptinal as a positive control inhibitor. **(M)** Bar graph demonstrates that ILA has no significant toxic effect on HIEC viability across tested concentrations.

This study employed network pharmacology to reveal the molecular mechanism of ILA in the treatment of intestinal injury in AP. Through the initial screening of the GeneCards database, 8,355 potential targets related to AP-induced intestinal injury were obtained, and by using the TargetNet and SwissTargetPrediction databases, 188 targets of ILA were screened out. Take the intersection by Venn diagram, and ultimately 157 common targets were obtained (Figure 6A). Through KEGG enrichment analysis, we found that the common targets were significantly enriched in cell connection regulation (gap junction/tight connection) pathway and the apoptotic regulation pathway (Figure 6C). Among them, genes such as CASP3, AKT1, and MMP9 present key node characteristics in the protein–protein interaction network (PPI) (Figure 6B). By screening apoptosis-related targets through molecular docking, it was found that CASP3 had the optimal binding energy with ILA (ΔG = –4.7 kcal/mol, Figure 6D). Moreover, ILA forms hydrogen bond interactions with the target proteins of CASP3, namely PHE-250, GLU-248, ASN-208, TRP-214, and GLU-246, to form a stable binding conformation (Figures 6E–G). By conducting CASP3 Western blotting experiments with IEC-18 lysis buffer treated by Drug Affinity Response Target Stability (DARTS) technology, researchers confirmed the interaction between ILA and CASP3 (Figures 6J,K). When further evaluating this interaction through the cell heat transfer assay (CETSA), it was found that, as expected, the incubation of ILA could significantly enhance the protein stability of CASP3 in the HIEC lysate under thermal denaturation conditions (Figures 6H,I).

To investigate the effect of ILA binding to CASP3 on its activity, further CASP3 activity tests were conducted. We found that ILA could inhibit the activity of CASP3 and had an inhibitory effect at an appropriate concentration (e.g., 10 μM) (Figure 6L). Further *in vitro* experiments revealed that ILA intervention could significantly inhibit LPS-induced apoptosis of HIEC (Figure 6M).

## Discussion

The gut microbiota, as an upstream regulatory factor for the occurrence and development of AP-related intestinal injury, has been widely recognized for its significance. Although the role of the gut microbiota and its bioactive products in the process of AP-related intestinal injury has attracted much attention, their specific impact on the development of AP-related intestinal injury is still not completely clear. Short-chain fatty acids like butyric acid derived from the gut microbiota may regulate inflammatory responses and lipid signaling pathways, thereby influencing the pathophysiology of acute pancreatitis (Wang et al., 2025). In addition to metabolic and dietary factors, pharmacological agents such as SGLT2 inhibitors have been reported to induce AP, highlighting the multifactorial nature of disease triggers (Li et al., 2025). Through 16S rDNA sequencing, we found that AKK were significantly downregulated in the intestines of AP model rats. Interestingly, through the clinical cohort of AP patients, we found that the relative abundance of AKK in the intestines of AP patients was significantly lower than that in the healthy population, and its relative abundance was negatively correlated with the levels of plasma inflammatory factors, C-reactive protein, and APACHE II score of the patients. We also found that live AKK and AKK culture supernatants could improve AP-related intestinal injury. Mechanism research has found that live AKK can biosynthesize ILA. Interestingly, ILA has demonstrated a protective effect on intestinal barrier function in all AP models. *In vitro* experiments further confirmed that ILA can specifically bind to CASP3 to inhibit LPS-induced apoptosis of intestinal epithelial cells and alleviate AP-related intestinal injury. The live AKK/ILA/CASP3 pathway identified in this study offers a novel perspective for the prevention and treatment of AP-related intestinal injury. Therefore, our data confirmed the role of live AKK and its bioactive products in alleviating AP-related intestinal injury and emphasized its potential therapeutic benefits for AP-related intestinal injury.

As the dominant symbiotic bacteria in the intestines of healthy people, the physiological abundance of AKK is usually maintained at 1.3–3.8% of the total flora (16S rDNA sequencing data) (Collado et al., 2007; Derrien et al., 2008). This study found that the intestinal AKK level of patients with AP-related intestinal injury was significantly lower than that of healthy people, and the decrease in its abundance might be related to the complex pathophysiology of AP. On the one hand, the decrease in the number of AKK may be related to AP. For example, the use of antibiotics leads to the disruption of the balance of intestinal microbiota (Becattini et al., 2016; Liu et al., 2024; McDonnell et al., 2021; Zimmermann and Curtis, 2019). On the other hand, AP itself may also lead to a decrease in the number of AKK. Because the abnormal activation of digestive enzymes and the influx of a large amount of inflammatory mediators will disrupt the intestinal microecological balance, thereby damaging the intestinal intima and changing the redox environment, which is not conducive to the growth of AKK (Xu et al., 2023; Wu et al., n.d.). In addition, the imbalance of intestinal microecology can also damage the mucus layer and mucin (an important energy source for AKK), further affecting the number of AKK (Ottman et al., 2017; Zhai et al., 2019). Our fecal transplantation experiments also indicated that a lower level of AKK in feces might aggravate intestinal injury in AP rats.

AKK, as a highly promising probiotic, have shown broad application prospects in the clinical treatment of various diseases. For example: AKK has demonstrated its beneficial activities in the fields of metabolic diseases, sepsis, cardiovascular diseases and immune-related colitis (Xie et al., 2023; Zhang et al., 2021; Lakshmanan et al., 2022; Chang et al., 2022), but there is still a significant knowledge gap in the molecular basis of its effect on improving the intestinal functional barrier. Through the microbiota transplantation experiment, our team found that live AKK demonstrated a significant protective effect on intestinal barrier function in the AP model. However, dead AKK have no protective effect on the intestinal barrier function. It is suggested that live AKK may produce certain bioactive substances to alleviate intestinal injury between AP. Further pretreatment with the supernatant of AKK solution revealed that rats pretreated with the supernatant of live AKK showed a significant protective effect on intestinal barrier function in the AP model. This suggests that the intestinal protective effect of AKK is closely related to its metabolic active products.

Biomarkers such as PCT, CRP, and TyG index have demonstrated utility in early diagnosis and severity stratification of AP, supporting the use of lipid profiles as additional indicators (Xinyu et al., 2025). Researchers found that disruptions in lipid metabolism through pathways such as PI3K/AKT/mTOR/SREBP1 have been shown to aggravate metabolic inflammation, similar to what may occur in severe acute pancreatitis (Chu et al., 2022). Given that the intestinal protective effect of AKK is closely related to its metabolites, we adopted non-targeted metabolomics and found that the content of ILA in the supernatant of AKK culture was significantly enriched compared with the control group. ILA has been confirmed to have a significant regulatory effect on intestinal homeostasis. We found in the AP rat model that ILA intervention demonstrated multi-dimensional therapeutic advantages. Not only by maintaining the polar distribution of ZO-1 protein to improve intestinal functional impairment. It can also reduce the levels of plasma IL-6 and TNF-*α* by significantly inhibiting the translocation of the intestinal flora.

To discover the targets of ILA, we screened out 188 targets of ILA from the TargetNet and SwissTargetPrediction databases. A total of 8,355 potential therapeutic targets for AP intestinal injury were initially screened from the GeneCards database. Finally, 157 public targets were obtained. Through KEGG enrichment analysis, we found that the common targets were significantly enriched in the intercellular junctions, tight junctions and apoptotic regulatory pathways. Among them, genes such as CASP3, AKT1, and MMP9 present key node characteristics in the protein–protein interaction network (PPI). Through molecular docking to screen apoptosis-related targets, we found that CASP3 had the optimal binding energy with ILA. The thermal shift assay (TSA) confirmed that the thermal stability of CASP3 was significantly improved after binding. *In vitro* experiments further confirmed that ILA intervention could significantly inhibit LPS-induced apoptosis of HIEC cells. This discovery reveals a novel paradigm of direct regulation of apoptotic executive proteins by microbial metabolites, and offers a new perspective that ILA shows better clinical translational potential due to its clear molecular target, compared with the traditional probiotic transplantation strategy for targeted treatment of AP intestinal injury. Our next steps will focus on establishing stable and reliable live bacteria, and ILA formulation processes. We will also complete systematic efficacy and safety evaluations in larger animal models. If the preclinical data continue to be excellent, we will plan to conduct initial clinical trials to assess its safety and initial efficacy for patients with acute pancreatitis.

Although this study has yielded meaningful findings, there are still several limitations. Although we have confirmed the protective effect of AKK and its metabolite ILA in the induction of intestinal injury by AP, the specific mechanism by which AKK regulates the production of ILA in the intestinal microenvironment has not yet been fully elucidated. Secondly, the sample sizes of animal experiments and clinical cohorts were still relatively small, which may affect the generalizability of the conclusions. In the future, larger sample sizes or multi-center data need to be used for verification. Furthermore, although *in vitro* and *in vivo* experiments have shown that ILA inhibits apoptosis by directly binding to CASP, its causal relationship still needs to be further verified through genetic experiments (such as the Casp3 gene knockout model). Finally, although ILA demonstrates therapeutic potential based on metabolites, its pharmacokinetic characteristics under the AP state, the optimal delivery strategy, and potential off-target effects still need to be systematically evaluated before clinical application. Solving these problems will provide an important foundation for promoting the clinical application of AKK and ILA.

## Data Availability

The data presented in this study are publicly available. The data can be found here: https://www.ncbi.nlm.nih.gov/sra, PRJNA1283475.

## Footnotes

1 https://pubchem.ncbi.nlm.nih.gov/

## References

[ref1] Becattini S. Taur Y. Pamer E. G. (2016). Antibiotic-induced changes in the intestinal microbiota and disease. Trends Mol. Med. 22, 458–478. doi: 10.1016/j.molmed.2016.04.003, 27178527 PMC4885777

[ref2] Chang C. C. Liu C. Y. Su I. C. Lee Y. J. Yeh H. J. Chen W. C. . (2022). Functional plasmon-activated water increases *Akkermansia muciniphila* abundance in gut microbiota to ameliorate inflammatory bowel disease. Int. J. Mol. Sci. 23:11422. doi: 10.3390/ijms231911422, 36232724 PMC9570201

[ref3] Chu H. Du C. Yang Y. Feng X. Zhu L. Chen J. . (2022). MC-LR aggravates liver lipid metabolism disorders in obese mice fed a high-fat diet via PI3K/AKT/mTOR/SREBP1 signaling pathway. Toxins (Basel). 14:833. doi: 10.3390/toxins14120833, 36548730 PMC9784346

[ref4] Collado M. C. Derrien M. Isolauri E. (2007). Intestinal integrity and *Akkermansia muciniphila*, a mucin-degrading member of the intestinal microbiota present in infants, adults, and the elderly. Appl. Environ. Microbiol. 73, 7767–7770. doi: 10.1128/AEM.01477-07, 17933936 PMC2168041

[ref5] Derrien M. Collado M. C. Ben-Amor K. Salminen S. de Vos W. M. (2008). The mucin degrader *Akkermansia muciniphila* is an abundant resident of the human intestinal tract. Appl. Environ. Microbiol. 74, 1646–1648. doi: 10.1128/AEM.01226-07, 18083887 PMC2258631

[ref6] Entezari M. Hashemi D. Taheriazam A. Zabolian A. Mohammadi S. Fakhri F. . (2022). AMPK signaling in diabetes mellitus, insulin resistance and diabetic complications: a pre-clinical and clinical investigation. Biomed. Pharmacother. 146:112563. doi: 10.1016/j.biopha.2021.112563, 35062059

[ref7] Goyal H. Awad H. Hu Z. D. (2017). Prognostic value of admission red blood cell distribution width in acute pancreatitis: a systematic review. Ann. Transl. Med. 5:342. doi: 10.21037/atm.2017.06.61, 28936436 PMC5599272

[ref8] Jung T. W. Park H. S. Choi G. H. Kim D. Lee T. (2018). β-Aminoisobutyric acid attenuates LPS-induced inflammation and insulin resistance in adipocytes through AMPK-mediated pathway. J. Biomed. Sci. 25:27. doi: 10.1186/s12929-018-0431-7, 29592806 PMC5875012

[ref9] Lakshmanan A. P. Murugesan S. Al Khodor S. Al Khodor S. (2022). The potential impact of a probiotic: *Akkermansia muciniphila* in the regulation of blood pressure-the current facts and evidence. J. Transl. Med. 20:430. doi: 10.1186/s12967-022-03631-0, 36153618 PMC9509630

[ref10] Li L. Li T. Liang X. Zhu L. Fang Y. Dong L. . (2025). A decrease in Flavonifractor plautii and its product, phytosphingosine, predisposes individuals with phlegm-dampness constitution to metabolic disorders. Cell Discov. 11:25. doi: 10.1038/s41421-025-00789-x, 40097405 PMC11914097

[ref11] Li R. Luo P. Guo Y. He Y. Wang C. (2025). Clinical features, treatment, and prognosis of SGLT2 inhibitors induced acute pancreatitis. Expert Opin. Drug Saf. 24, 1253–1257. doi: 10.1080/14740338.2024.2396387 39172128

[ref12] Liu S. Zhao S. Cheng Z. Ren Y. Shi X. Mu J. . (2024). *Akkermansia muciniphila* protects against antibiotic-associated diarrhea in mice. Probiotics Antimicrob Proteins 16, 1190–1204. doi: 10.1007/s12602-023-10101-6, 37314693

[ref13] Maheshwari R. Subramanian R. M. (2016). Severe acute pancreatitis and necrotizing pancreatitis. Crit. Care Clin. 32, 279–290. doi: 10.1016/j.ccc.2015.12.006, 27016168

[ref14] McDonnell L. Gilkes A. Ashworth M. Rowland V. Harries T. H. Armstrong D. . (2021). Association between antibiotics and gut microbiome dysbiosis in children: systematic review and meta-analysis. Gut Microbes 13, 1–18. doi: 10.1080/19490976.2020.1870402, 33651651 PMC7928022

[ref15] Ottman N. Davids M. Suarez-Diez M. Boeren S. Schaap P. J. dos Martins Santos V. A. P. . (2017). Genome-scale model and omics analysis of metabolic capacities of *Akkermansia muciniphila* reveal a preferential mucin-degrading lifestyle. Appl. Environ. Microbiol. 83:e01014–e01017. doi: 10.1128/aem.01014-17, 28687644 PMC5583483

[ref16] Percie du Sert N. Hurst V. Ahluwalia A. Alam S. Avey M. T. Baker M. . (2020). The ARRIVE guidelines 2.0: updated guidelines for reporting animal research. PLoS Biol. 18:e3000410. doi: 10.1371/journal.pbio.3000410, 32663219 PMC7360023

[ref17] Schietroma M. Pessia B. Carlei F. Mariani P. Sista F. Amicucci G. (2016). Intestinal permeability and systemic endotoxemia in patients with acute pancreatitis. Ann. Ital. Chir. 87, 138–144, 27179282

[ref18] Schmidt J. Rattner D. W. Lewandrowski K. Compton C. C. Mandavilli U. Knoefel W. T. . (1992). A better model of acute pancreatitis for evaluating therapy. Ann. Surg. 215, 44–56. doi: 10.1097/00000658-199201000-00007, 1731649 PMC1242369

[ref19] Szatmary P. Grammatikopoulos T. Cai W. Huang W. Mukherjee R. Halloran C. . (2022). Acute pancreatitis: diagnosis and treatment. Drugs 82, 1251–1276. doi: 10.1007/s40265-022-01766-4, 36074322 PMC9454414

[ref20] Wang C. Liu Z. Zhou T. Wu J. Feng F. Wang S. . (2025). Gut microbiota-derived butyric acid regulates calcific aortic valve disease pathogenesis by modulating GAPDH lactylation and butyrylation. iMeta 4:e70048. doi: 10.1002/imt2.70048, 40860435 PMC12371252

[ref21] Wu Z Xu Q Gu S Chen Y Lv L Zheng B . Akkermansia muciniphila Ameliorates Clostridioides difficile Infection in Mice by Modulating the Intestinal Microbiome and Metabolites. Front Microbiol. 13:841920. doi: 10.3389/fmicb.2022.841920 PMC9159907 35663882

[ref22] Xie S. Li J. Lyu F. Xiong Q. Gu P. Chen Y. . (2023). Novel tripeptide RKH derived from *Akkermansia muciniphila* protects against lethal sepsis. Gut 73, 78–91. 37553229 10.1136/gutjnl-2023-329996

[ref23] Xinyu X. Jiang Z. Qing A. Xinyu X. Jiang Z. Qing A. . (2025). Clinical significance of PCT, CRP, IL-6, NLR, and TyG index in early diagnosis and severity assessment of acute pancreatitis: a retrospective analysis. Sci Rep. 15:2924. 39849025 10.1038/s41598-025-86664-x PMC11758003

[ref24] Xu Y. Duan J. Wang D. Lihua L. Xiehong L. Lin Z. . (2023). *Akkermansia muciniphila* alleviates persistent inflammation, immunosuppression, and catabolism syndrome in mice. Meta 13:194. doi: 10.3390/metabo13020194, 36837813 PMC9961567

[ref25] Zhai Q. X. Feng S. S. Arjan N. Zhai Q. Feng S. Chen W. (2019). A next generation probiotic, *Akkermansia muciniphila*. Crit. Rev. Food Sci. Nutr. 59, 3227–3236. doi: 10.1080/10408398.2018.1517725, 30373382

[ref26] Zhang C. Li G. Lu T. Liu L. Sui Y. Bai R. . (2023). The interaction of microbiome and pancreas in acute pancreatitis. Biomolecules 14:59. 38254659 10.3390/biom14010059 PMC10813032

[ref27] Zhang J. Ni Y. Q. Qian L. L. Fang Q. Zheng T. Zhang M. . (2021). Decreased abundance of *Akkermansia muciniphila* leads to the impairment of insulin secretion and glucose homeostasis in lean type 2 diabetes. Adv. Sci. 8:2100536. 10.1002/advs.202100536 PMC8373164 34085773

[ref28] Zhao Y. Yang H. Wu P. Yang S. Xue W. Xu B. . (2024). *Akkermansia muciniphila*: a promising probiotic against inflammation and metabolic disorders. Virulence 15:2375555. doi: 10.1080/21505594.2024.2375555, 39192579 PMC11364076

[ref29] Zimmermann P. Curtis N. (2019). The effect of antibiotics on the composition of the intestinal microbiota - a systematic review. J. Infect. 79, 471–489. doi: 10.1016/j.jinf.2019.10.008, 31629863

