# Supplementary figures and images for: The metabolite ILA of Akkermansia muciniphila improves AP-related intestinal injury by targeting and inhibiting CASP3 activity
Source: Front Microbiol. 2025 Dec 5;16:1669383. doi: 10.3389/fmicb.2025.1669383 (PMC12715610; doi:10.3389/fmicb.2025.1669383)

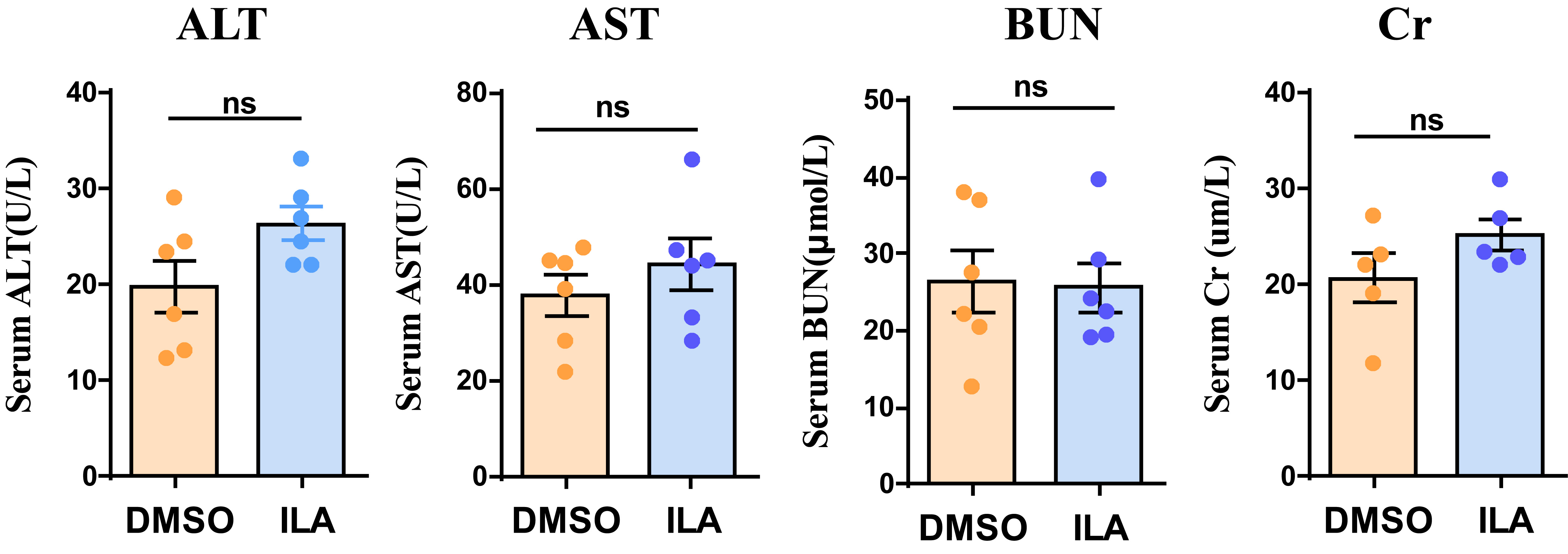

Supplement: Supplementary file 1 [file Image_1.jpeg]
